# Observations on Large Doses of Submuriate of Mercury for the Cure of Syphilis

**Published:** 1818-03

**Authors:** James Boyle

**Affiliations:** Assistant Surgeon, Royal Navy


### *Observations on large Doses of Submuriate of Mercury for the Cure of Syphilis.* By JAMES BOYLE, Assistant Surgeon, Royal Navy.

( Supernumerary to the East Indies. )

IN 1813, while an Hospital Mate at Haslar, I read a Work, entitled, "The Influence of Tropical Climates on European Constitutions," in which much was said in favour of calomel in *scruple doses* for the cure of tropical dysentery; the Author affirming that, in this way, the submuriate would be more likely to stop a purging than increase it. Judging from analogy, and knowing the effect of calomel *in small doses*, I confess that I suspected the accuracy of Mr. Johnson's statements, until I joined His Majesty's ship Spartan, in the latter end of 1813, where I found the Surgeon was occasionally in the habit of giving from fifteen to twenty grains of calomel as a purgative; but, contrary to that gentleman's expectations, the medicine almost invariably failed to produce catharsis, and was very generally followed by a confined state of the bowels and a soreness in the mouth, and occasionally some slight febrile symptoms.

Soon after this I joined His Majesty's ship Hydra, where I had an opportunity of treating successfully two cases of dysentery by administering scruple doses of calomel.

In 1814 I joined His Majesty's ship Benbow, in the West Indies, where dysentery prevailed to an alarming degree. The mercurial treatment, as described was adopted; and out of more than 400 cases of this disease, one only died. The general treatment and effects were as follow:—When a patient made his complaint known, a saline cathartic was administered, and on the same evening a scruple of calomel was given, after which the patient usually experienced an alleviation of the tormina and tenesmus. Should the mouth be sore the next day, which was sometimes the case,* the patient would complain of that only; but if the mouth was *not* affected, the dysentery continued, until by scruple doses ptyalism was established It seldom required more than three scruples to effect this object.

* One of the Editors, a few months ago, administered fifteen grains of calomel in conjunction with cathartics, in a threatening case of puerperal fever. The lady had nine copious stools, and yet in fifteen hours a smart ptyalism took place.

*Syphilis.* In very many cases of Syphilis, I have seen patients continue under a mercurial course for several weeks, without experiencing ptyalism, or any favourable change in the disease; but when, from any accidental circumstance, any of these patients happened to be confined to bed, in an apartment where the air was close, a violent salivation would suddenly break out, followed by the almost immediate healing of the chancres. Reflecting on these phænomena, I formed an idea that a single dose of mercury, if sufficiently large to induce ptyalism, might effect a cure in cases of recent venereal infection. The following instances, although they do not fully realize what I anticipated, may probably authorize a further trial of the plan proposed. One striking advantage of the scruple doses is, that while they ensure a more speedy developement of the *specific* mercurial influence on the system, by which *alone* the disease is checked in its progress, they are not so liable to act upon the bowels as small doses are, even when guarded by opium, and which thereby frustrate the real object in view, or greatly retard its accomplishment.

From what we observe in the appearance of chancres, there does not seem to be any check given to their enlargement, nor any amelioration effected in the acrid nature of their discharges, nor in the pain and irritation which they occasion, till the mouth is considerably sore. It follows from this, that all the time which elapses between the commencement of the medicine and the salivary excitation is time lost. Why then lose more than two or three days? The only objection that can be reasonably urged against the large doses of calomel, is the danger of violent *salivation* or violent *purging*. Now reiterated experience has proved the futility of the *latter* apprehension; and I believe, the *former* is equally groundless. For in the immense number of dysenteric cases, where we exhibited the calomel in scruple doses, there were scarcely any instances of very profuse or long-continued salivation. A mild ptyalism was generally the result, and the patient experienced no more inconvenience than where the same effect is produced by the common slow process. There is yet another consideration. We have fair reason to believe, that the *earlier* a chancre is attacked, the easier it is subdued, and the less necessity there is for *continuing* the mercurial action so long as it is generally continued. Indeed I may here remark, that most practitioners err, not only in not bringing on ptyalism *sooner*, but in keeping it up *longer* than is necessary. A certain time, however, appears to be requisite for the mercurial influence to exist after the healing of the chancre, and this very generally takes place after the scruple doses. In one case it continued seven days after the disappearance of the sore, or twenty-one from the time he left off the medicine.

As auxiliaries to the effect of mercury, the most rigid regimen should be enjoined, with warmth and confinement within doors. Much mischief is every day done—Many secondary symptoms owe their origin entirely to the neglect of these precautions.

His Majesty's Ship Prometheus,
Off Plymouth, September 3, 1817.

CASE 1. William Sprawton, marine, ætat. 28, entered the sick-list for the cure of well-marked venereal chancres, behind the corona glandis, with an accompanying bubo in the right groin. The sores were simply dressed, and a scruple of the submuriate of mercury administered. 4th. Had two evacuations in the night; mouth not the least affected; repeated the medicine to the same extent. At 7, P. M. mouth not affected; had one loose stool in the course of the day; repeated the medicine with the addition of five grains of the submuriate. 5th. Had no stool last night; mouth very slightly affected; repeated the mercury to a scruple: at 7, P. M. had no stool; no sensible alterations in the state of the mouth; repeated the medicine to gr. xv. 6th. A moderate ptyalism was now produced; has not had a stool for two days; discontinued the use of the mercury: Capiat solutio. magnes. sulph. ℥iv. At 7, P. M. had one stool to-day; sores have assumed a healing disposition; mouth a little more affected; bubo smaller; dressed the chancres with dry lint only. 7th. Had one stool in the night; mouth (as he said) still more affected; one of the chancres nearly healed. At 7, P. M. Bowels regular; no particular change in the state of the mouth. 8th. Had one evacuation in the night; no striking alteration in the chancres; bubo disappearing fast. 9th. Mouth much more affected; spits freely; bubo completely gone; had two stools in the night. 10th. Mouth continued well affected, chancres rather inflamed.* 11th. No particular change in the state of the mouth or sores. 12th, Mouth more affected; chancres less irritable; bowels regular. 13th.

* The patient was directed to keep quietly in his berth; as it was apprehended exercise in the open air might not only contribute to irritate the sores, but heal the mouth also.

Mouth, if any thing, more affected; sores appear greatly disposed to heal. 14th. One of the two chancres completely healed; the other much more superficial; bowels regular; mouth still considerably affected. 15th. Chancre healing rapidly; no other evident alteration in the symptoms. 17th. Mouth less painful; chancre not larger than a pin's head. 18th. Chancre scarcely perceptible, mouth not so considerably affected as it had been. 19th. Chancre completely healed. On the 26th of September this patient was discharged to duty in good health; his mouth being then completely well.

It may be proper to remark, that the patient on being interrogated after his discharge, was candid enough to acknowledge his having drunk spirituous liquors during the exhibition of the mercury; and to this I attributed the irritable appearance of the sores noticed in this case.

His Majesty's Ship Prometheus,
Off Weymouth, Nov. 10, 1817.

Case 2. Mr. —— an Officer in the Royal Navy, complained to me of a sore on the under and outer part of the prepuce, about the size of a three-penny silver piece, which he said made its appearance seventeen days previously to the above date, in the form of a small pustule; and which had now all the characteristic marks of a true venereal chancre. After a purgative had cleared his bowels, gave him hydrargyri submur. ℈j. 11th. Had one stool in the night; felt a disagreeable taste in his mouth; repeated the medicine to the same extent.* At 7, P. M. Had one stool in the course of the day; mouth a little tender; repeated the medicine. 12th. Had one stool in the night; mouth so painful as to be unable to eat bread; spits a little. 13th. Mouth very painful; spits freely; chancre assumes a healthy disposition. 14th. Mouth still more affected; bowels constipated. Habeat pulv. jalapæ ℈j. 15th. Mouth much affected; an evident diminution of size in the chancre; bowels regular. 16th. Mouth continues very much affected; bowels regular; chancre contracting rapidly. 17th, 18th, 19th, and 20th. Chancre completely healed; no particular change had taken place for the last four days, except the healing of the sore. The mouth still remains a little affected.

* This Officer was cured by three doses of medicine, and would not, I am convinced, have required so many, had he not for the greater part of the time, been exposed to the open air in the execution of his duty.

### Remarks by the Editors.

More than ten months ago, Mr. Cunningham, Surgeon of His Majesty's Ship Rochefort, informed one of the Editors of this Journal, that he had been, for a considerable time, in the habit of treating recent cases of chancre with scruple doses of calomel, by which means the cure was more rapidly and completely effected than by the usual plan. Upon the whole, we are of opinion that, in hospital practice particularly, the above proposal is highly deserving of a trial. In each of the two cases abovementioned, the mouth was sore on the third day, and the chancres healed on the tenth. This is a much quicker process than the common mode, which generally requires from three to six weeks.

